# Impact of warranty and green level of the product with nonlinear demand via optimal control theory and Artificial Hummingbird Algorithm

**DOI:** 10.1038/s41598-024-61453-0

**Published:** 2024-05-11

**Authors:** Hachen Ali, Fleming Akhtar, Amalesh Kumar Manna, Adel Fahad Alrasheedi, Ali Akbar Shaikh

**Affiliations:** 1 Department of Mathematics, The University of Burdwan, Burdwan, 713104 India; 2 Department of Mathematics, School of Applied Sciences, Kalinga Institute of Industrial Technology (KIIT), Deemed to be University, Bhubaneswar, 751024 India; 3 Department of Statistics and Operations Research, College of Science, King Saud University, P.O. Box 2455, 11451 Riyadh, Saudi Arabia

**Keywords:** Green manufacturing, Warranty policy, Carbon emission, Optimal control, Metaheuristic algorithms, Engineering, Mathematics and computing

## Abstract

Due to the current environmental situation and human health, a green manufacturing system is very essential in the manufacturing world. Several researchers have developed various types of green manufacturing models by considering green products, green investments, carbon emission taxes, etc. Motivated by this topic, a green production model is formulated by considering selling price, time, warranty period and green level dependent demand with a carbon emission tax policy. Also, the production rate of the system is an unknown function of time. Per unit production cost of the products is taken as increasing function of production rate and green level of the products. In our proposed model, carbon emission rate is taken as linear function of time. Then, an optimization problem of the production model is constructed. To validate of our proposed model, a numerical example is considered and solved it by AHA. Further, other five metaheuristics algorithms (AEFA, FA, GWOA, WOA and EOA) are taken to compare the results obtained from AHA. Also, concavity of the average profit function and convergence graph of different metaheuristics algorithms are presented. Finally, a sensitivity analysis is carried out to investigate the impact of different system parameters on our optimal policy and reach a fruitful conclusion from this study.

## Introduction

Providing a warranty gives customers peace of mind that the manufacturer stands behind the product's quality and will take care of any problems that crop up within the warranty period. To lower the possibility of warranty claims, manufacturers may raise the quality of their products, which will raise the standard of the product as a whole. Easy-to-use warranties that offer prompt service can increase patron happiness and loyalty. For manufacturers, servicing and warranty claims can be expensive, which has an impact on their bottom line. A well-run warranty programme, however, can also result in recurring revenue and favourable word-of-mouth recommendations, which can somewhat offset these expenses. A robust warranty programme can set a product apart from rivals and draw in buyers who appreciate the extra security.

On the other hand the environmental impact across their whole lifecycle, including the extraction of raw materials, production, usage, and disposal, products with a higher green level often have a reduced environmental impact. Reductions in pollution, greenhouse gas emissions, and resource usage may result from this. As people become more aware of environmental issues, they frequently see green products more favourably. Sales growth and enhanced brand loyalty may result from this. The environmental impact of items is governed by standards and laws in many different countries. Adhering to or surpassing these benchmarks can guarantee adherence to regulations and prevent sanctions. Because sustainable materials or production methods are used, creating and manufacturing green products may initially be more expensive. Nevertheless, over time, savings from less resource usage and waste disposal may be enough to balance these expenses. By drawing in eco-aware customers and setting a company apart from rivals, green products can give a business an advantage over rivals.

Apart from these, an optimal control problem is one in which an objective function is to be optimised by determining, under constraints, the optimal control inputs over time for a dynamical system. Finding the control technique that minimises or maximises the objective function while meeting any limitations and the dynamics of the system is the aim. Establish a quantifiable objective function to measure the system's performance. This could involve maximising profit, lowering the cost of control inputs, or bringing the system to the desired state. Use differential equations to explain the system's dynamics by connecting its state variables to its control inputs. Indicate any limitations on the state variables, the control inputs, or both. These limitations could be operational, safety-related, or physical in nature. Optimisation is the process of determining the control inputs to minimise or maximise the objective function while adhering to the limitations and dynamics of the system. This frequently entails applying strategies like calculus of variations, dynamic programming, or numerical optimisation methods to solve a mathematical optimisation problem. In the next section, we are going to discuss a brief literature review in details.

### Literature review

The term "environmental impact during the production process" describes the range of potential environmental consequences associated with manufacturing and other industrial processes. Resource consumption, pollution, waste production, and greenhouse gas emissions are just a few of the many variables that are included in these effects. Achieving sustainable and ecologically conscious production practices requires an understanding of these effects and their mitigation. The process of production frequently entails the extraction and use of natural resources like water, fossil fuels, and minerals. Ecosystem degradation, habitat destruction, and resource depletion can result from overuse of these resources. Large amounts of energy are needed for many production processes, and these energy sources are frequently non-renewable ones like coal, oil, or natural gas. By raising greenhouse gas emissions and air pollution, excessive energy use exacerbates climate change. The burning of fossil fuels and certain industrial processes release greenhouse gases (GHGs) like carbon dioxide (CO_2_) and methane (CH_4_). The greenhouse effect, which is made worse by these gases, is what causes global warming and climate change. The atmosphere may be exposed to pollutants from industrial processes, including particulate matter, sulphur dioxide (SO_2_), nitrogen oxides (NOx), and volatile organic compounds (VOCs). In addition to causing acid rain, air pollution can be harmful to ecosystems and human health. Pollutants from production processes have the potential to contaminate rivers, lakes, and oceans. Heavy metals, chemicals, and nutrient-rich materials are examples of common pollutants that can damage aquatic ecosystems by causing eutrophication. Waste products from the production process include solid waste, wastewater, and hazardous waste. Poor waste management can lead to pollution, contaminated soil, and negative impacts on the health of people and the environment. Chemicals are used in many production processes, some of which may be hazardous to people or the environment. Contamination of soil and water can result from the improper handling and disposal of hazardous chemicals. The well-being of humans and animals can be impacted by noise pollution caused by industrial production. Furthermore, the aesthetic value of natural areas can be adversely affected by the visual impact of industrial infrastructure, which can change landscapes. In this consequence, the pollution issue in the world changes the purchasing behaviour of customers who are conscious of their health and environment. In addition, manufacturing companies are changing their production strategies, which are based on customers’ purchasing behaviour and environmental pollution. Also, governments in developed countries are increasing the environmental awareness of producers and buyers to reduce environmental pollution. As a result, manufacturers, retailers, and stakeholders are increasing their green activities, such as green product production, low emissions of carbon, etc. Many researchers suggested different strategies for sustainable development and reducing environmental pollution for manufacturing companies. Among those, some interesting research has been reported here. Sangwan^1^ investigated the qualitative and quantitative advantages of green manufacturing through an experimental study on medium and small enterprises in India. Pirraglia and Saloni^2^ measured the environmental improvements by implementing green manufacturing in various companies in a fuzzy environment. Mittal et al.^3^ compared barriers and drivers to green manufacturing in Germany and India. Hu et al.^4^ analysed the social welfare and market effects of green activity in the manufacturing industry. Zhou et al.^5^ studied a case study on green manufacturing technologies in China to promote sustainable development. Rehman et al.^6^ observed the impact of greening policies on manufacturing organisations. A model of energy performance contracts for green manufacturing in China was developed by Liu et al.^7^. Ma et al.^8^ determined the pricing decisions with green manufacturing for substitutable items in supply chain management. He et al.^9^ investigated the impact of environmental regulation on the greening performance of manufacturing enterprises in China. Waheed et al.^10^ observed the impact of ecological behaviour of consumers on green manufacturing systems and innovation. Hassan and Jaaron^11^ considered quality management to enhance organisations' performance in green manufacturing. Yan et al.^12^ investigated the impact of green innovation in the manufacturing process with empirical evidence from OECD countries. D'Angelo et al.^13^ considered green investments for green activities in a manufacturing system for sustainable development. Also, they investigated the positive impact of green activities on economic performance. Barman et al.^14^ solved a three-layer supply chain problem with government subsidies. Ali et al.^15^ developed a defective green production system via optimal control theory and the Teaching Learning-Based Optimizer algorithm. Das et al.^16^ analysed an inventory model for green products with selling price and green level dependent demand considering payment strategy. Rahaman et al.^17^ developed a production model considering selling price, advertisement and green level dependent demand.

Customer demand may be significantly impacted by a product's warranty. In essence, a warranty is an assurance from the seller or manufacturer about the product's longevity, functionality, and quality. The conditions and terms of a warranty can affect how consumers feel about a brand overall and affect their decision to buy. A strong and comprehensive warranty gives customers assurances about the dependability and quality of a product. The assurance of a warranty serving as support for a product can allay worries regarding possible flaws or malfunctions, thereby stimulating demand. Customers can reduce their risk by using a warranty. It lowers the perceived risk of buying a product by guaranteeing that the manufacturer within a predetermined window of time will fix any problems. This decrease in perceived risk may have a favourable effect on demand. Providing a robust warranty enhances the perception of the brand. Businesses that have a reputation for dependability and client satisfaction are likely to stand behind their products with broad warranties. Demand can be increased and consumer attraction increased with a strong brand image. As a result, in the competitive business world, all manufacturing companies are fighting to increase their market demand, which maximises the manufacturer's profit. To pull and keep customers, several companies take up various measures that ensure product reliability for customers. However, the product warranty policy is very effective for electronics goods to enhance customers’ demand. Because the manufacturer needs to ensure the customer that the purchased product will perform smoothly during the product’s life cycle, Based on various aspects of warranty policy, several researchers suggested different disciplines. Vahdani et al.^18^ considered a free replacement policy during the warranty period for deteriorating items. Chang and Lin^19^ determined the optimal period of warranty and maintenance policy for the products of fixed life. Shahanaghi et al.^20^ optimised the strategy of preventive maintenance for two-dimensional extended warranty policies. Esmaeili et al.^21^ studied three levels of warranty policy impact in a manufacturer, customer, and agent coordination system. Liu et al.^22^ analysed the cost effect of a failure interaction-based multi-component system with a free warranty policy. Considering warranty and inspection policies, Sarkar and Saren^23^ studied an imperfect production system under inspection error. Chen et al.^24^ studied the impact of warranty and production periods with price-sensitive periods of warranty in a defective production system. Alqahtani and Gupta^25^ considered money-back guarantee and warranty policies in the sensor-embedded remanufacturing model under the strategy of preventive maintenance. Taleizadeh et al.^26^ solved a return and warranty policy-based production problem with imperfect production and product quality control with Hybrid NSGA-II. Guchhait et al.^27^ developed a setup cost reduction and quality improvement-based imperfect production model under shortages and warranty policies. Samanta and Giri^28^ studied warranty and price-sensitive demand in a supply chain model called the contract of cost sharing. Ruan et al.^29^ considered the learning effect policy to find the optimal preventive maintenance and warranty policies for new product design. Shang et al.^30^ designed maintenance and warranty policies for products under random working periods. Wang et al.^31^ investigated the impacts of inspection error and warranty cost on a supply chain model under different development modes. Das et al.^32^ developed a manufacturing system with warranty, free service and rework policy under emission taxation considering SAR sensitive demand.

Global industrialization increases rapidly with modern technology, which has a negative effect on the ecology, system, and environment because most industries and companies are focused on economic growth by reducing costs. As a result, humans and the environment are faced with various threats, like global warming, ozone destruction, toxic environments, etc. Day-to-day, the carbon emission rate increases in several sectors, viz., industries, transportation, AC, etc., which makes it an unsafe zone for humans and the ecological system. To reduce global warming, governments in developed countries are trying to control carbon emissions through carbon emission tax and regulation policies. Also, researchers have focused their attention on sustainability, the development of the environment, and controlling global warming. Anenberg et al.^33^ analysed the impacts of carbon emissions on human mortality and the quality of surface air in regional, sectoral, and global areas. Anenberg et al.^34^ investigated the co-benefits of health, global air quality, and climate change during the emission control of black carbon and methane. Alkhathlan and Javid^35^ examined the combined and individual impacts of Saudi Arabia's energy use, greenhouse gas emissions, and economic expansion. Boutabba^36^ addressed the impact of income, financial development, trade, and energy on carbon emissions in India. The advantages of reducing carbon emissions through different policies, assessments, monitoring, and technologies were studied by Huisingh et al.^37^. Dogan and Seker^38^ looked into how trade, financial development, and renewable and non-renewable energy affected carbon emissions in the nations with the highest renewable energy. Shuai et al.^39^ identified key factors of advantage and disadvantage of carbon emissions from the evidence of 125 countries from 1990 to 2011. Hanif^40^ conducted research and gave a presentation on how consumption of energy, both non-renewable and renewable, affects emissions and the rise in the price of carbon in Africa. Fan et al.^41^ developed a production inventory model by considering emission reduction investment under carbon cap and trade policy. Turki et al.^42^ solved an optimization problem under cap and trade policy with shortage facility. Jermsittiparsert and Chankoson^43^ addressed the tourism industry behaviour under the carbon emission and environmental threats situation by time series analysis in Thailand. Khan et al.^44^ examined the impact of environmental innovation and renewable energy on international trade as well as consumption-based carbon emissions in G7 nations. Hasan et al.^45^ studied an inventory model with a carbon tax, cap and trade policy and strict carbon limit regulations. Rout et al.^46^ investigated a supply chain model under carbon emission regulations. Ma et al.^47^ determined the optimal pricing strategy for automobile manufacturers under a reduction policy of carbon emissions and government intervention. Sarkar et al.^48^ developed a sustainable model on SCM (supply chain management) for fixed-lifetime items under improvement of production quality and carbon emissions. Lu and Sun^49^ developed a model by considering Carbon regulations, production capacity, and low-carbon technology level for new products. Dong et al.^50^ investigated the innovation of green technology and its impact on carbon emissions using evidence from developed countries. Astanti et al.^51^ solved a low carbon supply chained related problem with carbon cap and trade policy. Mishra^52^ constructed a supply chain model with carbon emission dependent demand under carbon tax and trade policy. Razzaq et al.^53^ analysed the effects of green innovation and tourism development on emissions of carbon and economic growth in the top ten GDP countries. Manna et al.^54^ solved a warranty and carbon emission reduction investment-based production problem by using meta-heuristic algorithms. Gao et al.^55^ studied a supply chain network design considering carbon cap and trade policy. Li et al.^56^ considered low carbon investment strategies in a closed loop supply chain with multiple carbon policies. A comparative overview of previous research that is relevant to our suggested work are presented in Table 1.

**Table 1:** Previous research pertaining to our suggested work.

Authors name with year	Types of model	Demand dependent on	Production rate	Green effect in the demand	Warranty period	Carbon emission
Guchhait et al.^57^	EOQ	Deterioration & credit period	–	No	No	No
San-José et al.^58^	EOQ	Constant	–	No	No	No
Hua et al.^59^	EOQ	Freshness level	–	No	No	Yes
Hemmati et al.^60^	EPQ	Stock and price	Constant	No	No	No
Chen et al.^24^	EPQ	Constant	Constant	No	Yes	No
Kundu & Chakrabarti^61^	EPQ	Constant	Constant	No	No	Yes
Giri et al.^62^	EPQ	Price, warranty & green level	Constant	Yes	Yes	No
Taleizadeh et al.^26^	EPQ	Uncertain demand	Constant	No	Yes	No
Saga et al.^63^	EPQ	Stochastic demand	Constant	No	Yes	No
Mondal & Giri^64^	Supply chain	Deterministic	–	Yes	No	No
Manna et al.^65^	EPQ	warranty period and Selling price discount	Constant	No	Yes	No
Guchhait et al.^27^	EPQ	Constant	Constant	No	Yes	No
Hou et al.^66^	EPQ	Constant	Constant	No	Yes	No
Qu et al.^67^	Supply chain	Price & warranty	–	No	Yes	Yes
Manna et al.^68^	EPQ	Warranty period and emission level	Constant	No	Yes	Yes
Keshavarz-Ghorbani&ArshadiKhamseh^69^	Supply chain	Warranty length & price sensitive	–	No	Yes	No
Paul et al.^70^	EOQ	Price and green level	–	Yes	No	Yes
Ali et al.^15^	EPQ	Price, time & green level	Unknown function of time	Yes	No	No
Das et al.^71^	EPQ	Price, time & green level	Unknown function of time	Yes	No	Yes
This work	EPQ	Selling price, time, warranty & green level of the product	Unknown function of time	Yes	Yes	Yes

In this work, a non-linear market demand and carbon emission tax policy-based green manufacturing model is developed. The major aim of this work is the sustainable development of the environment as well as manufacturing companies by producing green products and reducing carbon emissions. For this purpose, green item production and taxation on carbon emissions are considered in this model. In addition, time, the product’s green level, the period of warranty, and price-sensitive market demand are assumed. Moreover, unit production cost is considered production rate and level of the product’s greenness dependent, whereas rate of emission of carbon is a linear function of time during the production. The following interrogatories of the raised model are inquired:

(i) What would be the optimal level of greenness for a product to maximise profit for the manufacturer?
(ii) How the average profit of the system is impact due to the maintenance of the level of greenness of an items?
(iii) What would be the production rate of the manufacturer for maximizing the profit of the system under payable carbon tax?
(iv) How the optimal policy of the system is affected due to provide warranty policy of the products to the customers?
(v) In order to maximise the manufacturer's profit, what would be the best selling price for the product?
(vi) The production model we have proposed poses a highly non-linear optimisation problem. How can this be resolved?
(vii) In order to maximise the manufacturer's profit, what would be the ideal time of year?

To get the answers to the above queries, a maximisation problem is constructed corresponding to the manufacturer’s average profit, which is highly non-linear. Then solve the said maximisation problem using the AHA, AEFA, EOA, FA, GWOA, and WOA algorithms and the theory of optimal control. Finally, statistical and post-optimality analyses are performed in order to check the feasibility of the optimal solution of the proposed model.

Therefore, the primary contributions to the work we have proposed are provided by

(i) A production system is considered where customers’ demand rate is dependent on green level of the products, selling price, time and warranty period of the products.
(ii) Manufacturers might think of their production rate as a dynamically time-dependent function, which means that it changes over time in reaction to different circumstances. Among other things, these variables may be shifts in demand, equipment failures, maintenance plans, raw material availability, and labour availability. By representing the production rate as a dynamic function, manufacturers can enhance their comprehension and control over their manufacturing procedures. Manufacturers can effectively respond to changes in market conditions or internal factors impacting production, optimise production plans, and allocate resources efficiently by taking into account the dynamic character of production rates. As a results, production rate of the manufacturer is an unknown function of time.
(iii) Per unit production cost of the produced items is a linear function of production rate and nonlinear function of green level of the products.
(iv) Manufacturers normally reserve a certain sum when they issue a warranty on their products in order to pay for any future claims. A warranty reserve or provision is what this sum is referred to be. In order to guarantee that it has the money on hand to carry out its warranty duties, such fixing or replacing damaged goods, the manufacturer invests this sum. The anticipated rate of product failures, the cost of repairs or replacements, and the length of the guarantee period are some of the variables that determine how much of a reserve is needed for warranties. Manufacturers can control their financial risk and guarantee they can fulfil their warranty commitments without materially affecting their profitability by allocating and investing a suitable amount for warranties. Hence the manufacturer needs invests an amount due to the maintenances of the warranty of the products.
(v) A manufacturer is obligated to pay the government a fee determined by the quantity of greenhouse gases (such as carbon dioxide) or other emissions they produce during manufacturing, which is known as a carbon emission tax. It is common practice to describe the rate of carbon emissions during production as a linear function of time, implying that emissions rise or fall at a steady pace over time. The producer would multiply the emissions rate by the tax rate per unit of emissions to get the amount of carbon emission tax due. Governments hope to encourage firms to adopt more ecologically friendly production practices and lessen their carbon impact by enacting a levy on carbon emissions. The manufacturer is required to pay the government a carbon emission tax. The rate of carbon emissions during production is represented as a linear function of time.
(vi) We have employed six well known meta-heuristics algorithms (Artificial Hummingbird Algorithm (AHA) (Zhao et al.^72^), Grey Wolf Optimizer Algorithm (GWOA) (Mirjalili et al.^73^), Equilibrium Optimizer Algorithm (EOA) (Faramarzi et al.^74^), Artificial Electric Field Algorithm (AEFA) (Yadav et al.^75^), Firefly Algorithm (FA) (Yang & He^76^) and Whale Optimizer Algorithm (WOA) (Mirjalili and Lewis^77^) to solve our optimization problem which is highly nonlinear in nature.

The manuscript is organised is as follows: Assumptions and notation that have been used throughout the paper are presented in Sect. "Notation and assumptions". Section "Problem description and mathematical formulation" provides our proposed model's mathematical formulation, while Sect. "Solution methodology" presents the solution methodology of our proposed model. In Sect. "Numerical analysis", we have discussed the optimization method (brief description of the Artificial Hummingbird Algorithm (AHA) (Zhao et al.,^72^). In Sect. "Sensitivity Analysis", a numerical example is considered to validate our model and solved it using different metaheuristics algorithms. Also, statistical experiment, ANOVA test, convergence graph of different algorithms and concavity graph of average profit function are presented in Sect. "Sensitivity Analysis". Finally, in Sect. "Managerial insights", sensitivity analysis is performed in order to reach the fruitful conclusion of this study.

## Notation and assumptions

### Notation

See Abbreviations section.

### Assumptions

(i) Planning and controlling production becomes more dynamic when the rate of production is taken as an unknown function of time. This recognises that there will always be variances, uncertainties, and changes in production processes. A business's ability to make decisions and manage operations effectively depends on its ability to recognise the significance of this variability. Here, a production system is considered where rate of production of the manufacturer $M\left( t \right)$ is an unknown function of time.
(ii) Important elements that have a big influence on customer demand are a product's selling price, green level and warranty. Every single one of these factors has an impact on decisions about what to buy. Often, the selling price is the most obvious and immediate factor affecting consumer demand. Value for money and affordability are directly impacted. Consumers typically seek after products that offer a balance between quality and price. Demand can be increased and a wider customer base drawn in with a competitive and fair price. Different market segments have differing levels of price sensitivity, so companies must carefully position their products to appeal to their target market. Beside the selling price of the product, environmental awareness has grown to be a major factor influencing consumer behaviour in recent years. The eco-friendliness or "green level" of a product has become more and more important. There is a growing trend among consumers to make decisions that support ecologically friendly and sustainable practices. A growing number of consumers are becoming environmentally conscious, and products with less of an impact on the environment—like those made of recycled materials or with energy-efficient features—often appeal to them. Businesses that implement sustainable practices may also draw in eco-aware customers and improve their brand image. Finally, A product's warranty can have a big impact on how confident and trusting customers are of a brand. A strong warranty shows that the manufacturer is confident in the longevity and quality of the product. Consumers frequently see longer or more comprehensive warranties as signs of reliability and investment value. This can reduce worries about possible flaws or malfunctions, which will increase demand. In addition to providing a competitive edge, a strong warranty also shows the manufacturer's dedication to providing excellent customer service, which can increase customer satisfaction. Combining all the above mentioned factor, here it is assumed the demand rate,$$D_{1} \left( {s_{p} ,t,\theta_{g} ,t_{w} } \right) = d_{0} - d_{1} s_{p}^{a} + d_{2} t + d_{3} \theta_{g}^{b} + d_{4} t_{w}^{c} \,{\text{where}}$$$$d_{0} ,d_{1} ,d_{2} ,d_{3} ,d_{4} ,a,b,c > 0$$ is considered nonlinear function of green level, selling price, warranty time and a linear function of time.
(iii) Modern business strategies that seek to strike a balance between environmental responsibility and economic efficiency must take into account the relationship between production costs and the green level, or environmental sustainability. Companies looking to reduce their environmental effect while keeping costs low must comprehend this relationship. In this research, manufacturer per unit production cost of the items is taken as a linear function of $M\left( t \right)$ and also it is dependent on the green level of the product. Manufacturer per unit production cost of the items is presented by the following expression:
  $$c_{p} \left( {M\left( t \right),\theta_{g} } \right) = \lambda_{1} + \lambda_{2} M\left( t \right) + \lambda_{3} \theta_{g}^{d}$$ where $$\lambda_{1} ,\lambda_{2} ,\lambda_{3} ,d > 0.$$
(iv) Companies use warranty cost investment as a tool for financial planning to make sure they can fulfil their warranty obligations to customers. It demonstrates a dedication to quality and client fulfilment while also controlling the financial risks related to warranty claims and repairs. Manufacturer must invest an amount due to warranty of demand product and per unit warranty cost is constant and it is given by $w_{c}$.
(v) A carbon tax is a tool used by policymakers to try and internalise the external costs associated with carbon emissions in an effort to cut greenhouse gas emissions and slow down global warming. The significance of a carbon tax lies in its ability to address the environmental, social, and economic issues associated with climate change. Because of the carbon emissions they produce during the production process, manufacturers are required to pay a carbon emission tax to the government. Here, it is assumed that the tax rate on carbon emissions from manufacturers is linearly dependent on time. This is expressed mathematically as follows
  $$\theta_{m} \left( t \right) = b_{1} + b_{2} t,\,\,\,\,\,\,0 \le t \le T.$$
  $${\text{where}}\,\,b_{1} ,\,b_{2} > 0\,.$$
(vi) Time horizon is finite for this proposed model.
(vii) Replacements/shortages are not allowed in this model and lead time is constant.

## Problem description and mathematical formulation

**Figure 1:**
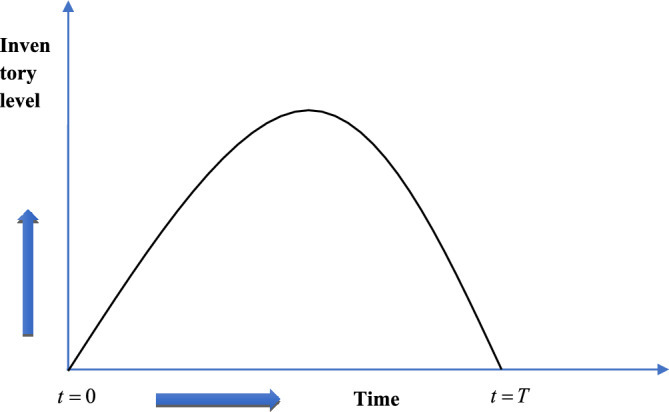
Graphical representation of inventory level with respect to time.

In this section, we have formulated a production inventory model based on the assumption made in sub-section "Assumption" and with the help of the notations mentioned in sub-Sect. "Notation". The manufacturing company generated product at the rate $M\left( t \right)$ during a specific time period in the suggested inventory production system. Thereafter the stock level of the manufacturer declines due to meet up the customers’ demand rate and at $t = T$, it becomes zero. Graphical representation of the system is presented in Fig. 1.

The following differential equation represents the current inventory level status for the suggested production inventory model.

1 $$\frac{dx\left( t \right)}{{dt}} = \,M\left( t \right) - D_{1} \left( {s_{p} ,t,\theta_{g} ,t_{w} } \right), \, 0 \le t \le T$$

with the boundary conditions $x\left( t \right) = x\left( T \right) = 0$.

Sales revenue and different cost component of the manufacturer are calculated as follows:

(i) The amount of all money received from the sale of goods or services over a given time period is the total sales revenue throughout the life of a product or business venture. Depending on the situation, this time frame may change according to a manufacturing cycle, or a product's lifecycle. The total sales revenue is
  2 $$\begin{aligned} SR\left( {s_{p} ,\theta _{g} ,T} \right)\, & = \int_{0}^{T} {\, s_{p} } D_{1} \left( {s_{p} ,t,\theta _{g} ,t_{w} } \right)\, dt\, \, \\ & = s_{p} T\left( {d_{0} - d_{1} s_{p}^{a} + \frac{{d_{2} T}}{2} + d_{3} \theta _{g}^{b} + d_{4} t_{w}^{c} } \right) \\ \end{aligned}$$
(ii) The average cost per claim multiplied by the anticipated number of warranty claims determines the product's overall warranty cost. One can estimate the anticipated number of warranty claims using engineering study, industry norms, or historical data. It takes into consideration variables such the product's failure rate, warranty duration, and usage trends. Manufacturers can budget for warranty costs, set aside money for warranty reserves, and assess the overall cost-effectiveness of their warranty programmes by computing the total warranty cost. The total warranty cost of the demand product is calculated as:
  3 $$\begin{aligned} WC\left( {s_{p} ,\theta_{g} ,T} \right)\, & = \int_{0}^{T} {\,w_{c} } D_{1} \left( {s_{p} ,t,\theta_{g} ,t_{w} } \right)\,dt \\ \, & = w_{c} \left( {d_{0} - d_{1} s_{p}^{a} + \frac{{d_{2} T}}{2} + d_{3} \theta_{g}^{b} + d_{4} t_{w}^{c} } \right)T. \\ \end{aligned}$$
(iii) The total production cost is
  4 $$PC\left( {s_{p} ,\theta_{g} ,T} \right) = \,\int_{0}^{T} {c_{p} \left( {M\left( t \right),\theta_{g} } \right)} \,M\left( t \right)dt = \int_{0}^{T} {\left( {\lambda_{1} + \lambda_{2} \,M\left( t \right) + \lambda_{3} \theta_{g}^{d} } \right)M\left( t \right)dt}$$
(iv) The total holding cost is presented by
  5 $$HC\left( T \right) = h_{c} \int\limits_{0}^{T} {x\left( t \right)dt}$$
(v) Total tax paid to the government for carbon emission is calculated as:
  6 $$CET\left( T \right) = \int_{0}^{T} {c_{m} \left( {b_{1} + b_{2} t} \right)dt} = \left[ {c_{m} \left( {b_{1} + \frac{{b_{2} T}}{2}} \right)T} \right]$$

From (2), (3), (4), (5) and (6), The manufacturer's overall profit for a cycle is calculated by deducting the total revenue from sales from the total cost of manufacturing. The selling price per unit multiplied by the total number of units sold yields the total revenue. It stands for the entire revenue received from product sales during the cycle.

The whole cost of a product comprises all of the costs associated with its manufacturing and sale, including labour, raw materials, overhead, and other operating expenses. If appropriate, the cost of warranty claims is also included. Hence,

Total profit = Total revenue—Total cost.

Therefore, the total profit of the manufacturer per cycle is given by

7 $$\begin{aligned} TP_{1} \left( {s_{p} ,\theta _{g} ,T} \right)\, & = \left( {SR\left( {s_{p} ,\theta _{g} ,T} \right) - PC\left( {s_{p} ,\theta _{g} ,T} \right) - HC\left( T \right) - WC\left( {s_{p} ,\theta _{g} ,T} \right) - CET\left( T \right) - A} \right) \\ & = \left( {s_{p} - w_{c} } \right)\left( {d_{0} - d_{1} s_{p}^{a} + \frac{{d_{2} T}}{2} + d_{3} \theta _{g}^{b} + d_{4} t_{w}^{c} } \right)T - A \\ & - \int_{0}^{T} {\left\{ {\left( {\lambda _{1} + \lambda _{3} \theta _{g}^{d} } \right)M\left( t \right) + \lambda _{2} M^{2} \left( t \right)} \right\}\, dt} - h_{c} \int\limits_{0}^{T} {x\left( t \right)dt} - c_{m} T\left( {b_{1} + \frac{{b_{2} T}}{2}} \right) \\ \, & = \left\{ {\left( {s_{p} - w_{c} } \right)\left( {d_{0} - d_{1} s_{p}^{a} + \frac{{d_{2} T}}{2} + d_{3} \theta _{g}^{b} + d_{4} t_{w}^{c} } \right)T - c_{m} T\left( {b_{1} + \frac{{b_{2} T}}{2}} \right) - A} \right\} \\ & - \int_{0}^{T} {\left\{ {\left( {\lambda _{1} + \lambda _{3} \theta _{g}^{d} } \right)M\left( t \right) + \lambda _{2} M^{2} \left( t \right) + h_{c} x\left( t \right)} \right\}\, dt} \\ \end{aligned}$$

Therefore, average profit of the manufacturer can be written as:

8 $$AP_{1} \left( {s_{p} ,\theta_{g} ,T} \right) = \frac{{TP_{1} \left( {s_{p} ,\theta_{g} ,T} \right)}}{T}$$

Here, our objective is to solve the following optimization problem:

9 $$\left\{ {\begin{array}{*{20}c} {Maximize\, } & {AP_{1} \left( {s_{p} ,\theta _{g} ,T} \right)} \\ {Subject\, \, \,to\, } & {s_{p} > 0,\, 0 < < \theta _{g} \le 1,\, T > 0} \\ \end{array} } \right.$$

## Solution methodology

First, we have to solve the following optimal control problem to find the solution of the optimization problem (9)

$$Minimize\,\,V = \frac{1}{T}\int_{0}^{T} {\left\{ {\left( {\lambda_{1} + \lambda_{3} \theta_{g}^{d} } \right)M\left( t \right) + \lambda_{2} M^{2} \left( t \right) + h_{c} x\left( t \right)} \right\}\,dt}$$

10 $$\begin{gathered} Subject \, to \, the \, conditions \hfill \\ \begin{array}{*{20}c} \begin{gathered} \frac{dx\left( t \right)}{{dt}} = M\left( t \right) - D_{1} \left( {s_{p} ,t,\theta_{g} ,t_{w} } \right) \hfill \\ and\,x\left( 0 \right) = x\left( T \right) = 0 \hfill \\ \end{gathered} \\ {\,\,\,\,\,\,\,\,\,\,\,\,\,} \\ \end{array} \hfill \\ \end{gathered}$$

A mathematical optimisation problem known as an optimal control problem is one in which the goal is to maximise a given objective function by determining the optimal control strategy for a dynamical system. Put another way, it looks for the best approach to gradually adjust a system's controls in order to get the intended result. By eliminating the value of $M\left( t \right)$ in the optimal control problem (10), it takes the form given below:

11 $$\begin{gathered} Minimize\,\,V \hfill \\ = \frac{1}{T}\int_{0}^{T} {\left\{ {\left( {\lambda_{1} + \lambda_{3} \theta_{g}^{d} } \right)\left( {\dot{x}\left( t \right) + D_{1} \left( {s_{p} ,t,\theta_{g} ,t_{w} } \right)} \right) + \lambda_{2} \left( {\dot{x}\left( t \right) + D_{1} \left( {s_{p} ,t,\theta_{g} ,t_{w} } \right)} \right)^{2} + h_{c} x\left( t \right)} \right\}dt} \hfill \\ = \int_{0}^{T} {H_{1} \left( {t,x\left( t \right),\dot{x}\left( t \right)} \right)\,dt} \hfill \\ \end{gathered}$$

$$Subject \, to \, \,x\left( 0 \right) = \,x\left( T \right) = 0$$

where Hamiltonian of the system is

$$H_{1} \left( {t,x\left( t \right),\dot{x}\left( t \right)} \right) = \frac{1}{T}\left\{ {\left( {\lambda_{1} + \lambda_{3} \theta_{g}^{d} } \right)\left( {\dot{x}\left( t \right) + D_{1} \left( {s_{p} ,t,\theta_{g} ,t_{w} } \right)} \right) + \lambda_{2} \left( {\dot{x}\left( t \right) + D_{1} \left( {s_{p} ,t,\theta_{g} ,t_{w} } \right)} \right)^{2} + h_{c} x\left( t \right)} \right\}$$

### Theorem 1.

The system's inventory level is determined by

12 $$x\left( t \right) = \left[ {\left( {\frac{{d_{2} }}{2} - \frac{{h_{c} }}{{4\lambda_{2} }}} \right)t\left( {T - t} \right)} \right],\,\,\,0 \le t \le T.$$

### Proof.

Since, optimal control problem (10) is equivalent to the variational problem (11) so, it is sufficient to solve (11). Now, applying Euler-Lagrange equation to (11), we get,

$$\frac{{\partial H_{1} }}{\partial x} - \frac{d}{dt}\left( {\frac{{\partial H_{1} }}{{\partial \dot{x}}}} \right) = 0\,\,\,or,\frac{{d^{2} x}}{{dt^{2} }} = \frac{{h_{c} }}{{2\lambda_{2} }} - d_{2}$$

Using the condition, $x\left( t \right) = x\left( T \right) = 0$, the stock level at time $t$ is given by

$$x\left( t \right) = \left( {\frac{{d_{2} }}{2} - \frac{{h_{c} }}{{4\lambda_{2} }}} \right)\left( {T - t} \right)t,\,\,\,\,0 \le t \le T.$$

### Theorem 2.

Production rate $M\left( t \right)$ is determine in the following way

13 $$M\left( t \right) = \left\{ {\left( {\frac{{d_{2} }}{2} - \frac{{h_{c} }}{{4\lambda_{2} }}} \right)\left( {T - 2t} \right) + \left( {d_{0} - d_{1} s_{p}^{a} + d_{2} t + d_{3} \theta_{g}^{b} + d_{4} t_{w}^{c} } \right)} \right\}\,\,\,\,\,\,\,\,\,\,\,0 < t \le T.$$

### Proof:

From Eq. (1) one can easily get

$$P\left( t \right) = \frac{dx\left( t \right)}{{dt}} + D_{1} \left( {s_{p} ,t,\theta_{g} ,t_{w} } \right)$$

Now from Eq. (12) one can obtained

$$\frac{dx\left( t \right)}{{dt}} = \left( {\frac{{d_{2} }}{2} - \frac{{h_{c} }}{{4\lambda_{2} }}} \right)\left( {T - 2t} \right)$$

Putting the value of $\frac{dx\left( t \right)}{{dt}}$ and $D_{1} \left( {s_{p} ,t,\theta_{g} ,t_{w} } \right)$ we get

$$P\left( t \right) = \left\{ {\left( {\frac{{d_{2} }}{2} - \frac{{h_{c} }}{{4\lambda_{2} }}} \right)\left( {T - 2t} \right) + \left( {d_{0} - d_{1} s_{p}^{a} + d_{2} t + d_{3} \theta_{g}^{b} + d_{4} t_{w}^{c} } \right)} \right\}$$

This completes the proof.

### Corollary 1.

The holding cost is calculated as

14 $$HC\left( T \right) = \frac{{h_{c} T^{3} }}{6}\left( {\frac{{d_{2} }}{2} - \frac{{h_{c} }}{{4\lambda_{2} }}} \right)$$

### Corollary 2.

The production cost is as follows

15 $$\begin{gathered} \,\,\,\,\,PC\left( {s_{p} ,\theta_{g} ,T} \right) \hfill \\ = \left\{ \begin{gathered} \left( {\lambda_{1} + \lambda_{3} \theta_{g}^{d} } \right)T\left( {K_{2} T + \frac{{K_{1} T^{2} }}{2}} \right) \hfill \\ \, + \lambda_{2} \left( {K_{2}^{2} T + K_{1} K_{2} T^{2} + \frac{{K_{1}^{2} T^{3} }}{3}} \right) \hfill \\ \end{gathered} \right\} \hfill \\ {\text{where, }}K_{1} = \frac{{h_{c} }}{{2\lambda_{2} }}{\text{ and }}K_{2} = d_{0} - d_{1} s_{p}^{a} + d_{3} \theta_{g}^{b} + d_{4} t_{w}^{c} + \left( {\frac{{d_{2} }}{2} - \frac{{h_{c} }}{{4\lambda_{2} }}} \right)T. \hfill \\ \end{gathered}$$

### Problem 1.

The corresponding optimization problem of the system can be written as:

16 $$\left\{ \begin{gathered} Maximize\,\,AP_{1} \left( {s_{p} ,\theta_{g} ,T} \right) = \frac{{SR\left( {s_{p} ,\theta_{g} ,T} \right) - WC\left( {s_{p} ,\theta_{g} ,T} \right) - CET\left( T \right) - HC\left( T \right) - PC\left( {s_{p} ,\theta_{g} ,T} \right) - A}}{T} \hfill \\ Subject\,\,to\,\,s_{p} > 0,\,0 < < \theta_{g} \le 1,\,T > 0 \hfill \\ \end{gathered} \right.$$

### Optimization method

The average function $AP_{1} \left( {s_{p} ,\theta_{g} ,T} \right)$ for the problem (16) is non-linear and complicated w. r. to decision variables viz. price $\left( {s_{p} } \right),$ green level of the product $\left( {\theta_{g} } \right)$ and cycle length $\left( T \right)$ that indicates a complexity to find a closed form solution of (16). The Artificial Hummingbird Algorithm (AHA (Zhao et al.^72^) has been utilised as a tool to help us find a solution to the problem that is either optimal or very close to the optimal solution from Eq. (16). AHA has just developed one of the most popular metaheuristic algorithms. The following are some of the reasons why AHA algorithms should be used to solve the suggested model:

- It is a newly created algorithm.
- This algorithm is something different from the existing algorithm.
- It is very much efficient algorithm for solving the problem.
- Because of AHA's memory capacity, flight skill, and foraging techniques, it differs greatly from previous metaheuristic algorithms in that it is biologically grounded.

The other algorithms drastically alter when the aforementioned factors are taken into account. Three foraging techniques and three flight motions used by hummingbirds serve as the foundation of AHA. The flight ability, memory capacity, and foraging strategies of this algorithm are its main advantages. The AHA method's computing complexity is affected by initialization, fitness evaluation, hummingbird position update, population size, maximum number of iterations, and variable dimension. The following diagram illustrates how computationally complex AHA is overall:

$$\begin{gathered} Order(AHA) = Order\,(problem\,definition) + Order\,(initilization) + Order\,(t(\,evaluation\,of\,function) \hfill \\ \,\,\,\,\,\,\,\,\,\,\,\,\,\,\,\,\,\,\,\,\,\,\,\,\,\,\, + Order(t(guided\,foraging)) + Oerder(t(territorial\,foraging)) + Order(t(migration\,foraging)) \hfill \\ \end{gathered}$$

The three main parts of AHA are listed below:

#### Sources of food

In reality, before choosing from a variety of food sources, an HB evaluates source characteristics such as nectar quality and refilling rates, particular flowers, and the most recent time it visited the blossoms. Each food source in AHA is assumed to have the same kind and quantity of flowers; a food source is seen as a solution vector, and the rate at which a nectar (i.e., food source) replenishes itself is regarded as a fitness value. The rate of food source replenishment will increase with fitness level.

#### Hummingbirds (HB)

Each HB is given a certain nectar to eat on, and the nectar and the HB are both in the same area. A HB is able to recall and share with other HBs in the community the location and rate of nectar replenishment of a single food source. Additionally, each HB may remember the last time it visited a certain food source.

#### Visit table

The table of visits, which displays the interval since the same HB last visited that food source, reports the degree of visit for each nectar for each particular HB. An HB's top visit priority will be to a food source with a high visit level. An HB will go to the food source that has the highest rate of refilling a nectar among the sources in order to obtain more nectar. The visit table can be used by each HB to identify its favourite food source. The table is modified after each loop.

AHA is a metaheuristic algorithm for solving optimisation problems which is developed on the behaviour of the bird (Hummingbird). This section presents three mathematical models that simulate directed foraging, territorial foraging, and migratory foraging in hummingbirds. Figure 3 depicts the three foraging behaviour. The suggested algorithm follows the same three basic stages as the majority of swarm-based optimizers in terms of their structure. The AHA is summarised in Algorithm 1.
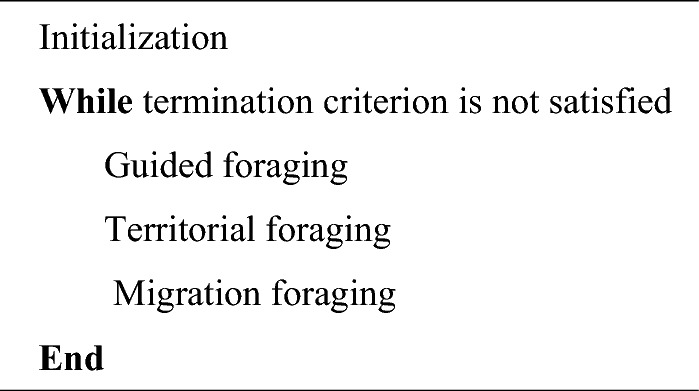


### Initialization

The following is a random initialization of a population of *n* HBs on *n* food sources.

$$u_{i} = lower + r(upper - lower)$$

where *lower* and *upper* be the lower boundary and upper boundary of a *d* dimensional search space, *r* be the random number lies in [0,1], $u_{i}$ be the position of *i*-th (*i* = *1, 2…, n*) and which is the original solution of the problem.

The food source is also initialized in the following way

$$FS_{{i,j}} = \left\{ {\begin{array}{*{20}l} {null} & {if\, i = j} \\ 0 & {if\, \, \, \, i \ne j} \\ \end{array} } \right.\, \,\quad i = 1,2,...,n\, ;\, \, j = 1,2,...,n$$

For $$FS_{i,j} = null$$$$if\,i = j$$, it implies that an HB eats from a nectar and $$FS_{i,j} = 0$$$$if\,i \ne j$$ implies that the source of *j*-th source just visited by *i-*th HB.

### Guided foraging

Each HB has a built-in urge to look at the nectar in the highest quantity, therefore the supply must replenish nectar quickly and wait a long time between HB visits. Finding food sources with the highest percentage of foraging-guided behavioural visits is advised by the AHA for HBs. AHA then suggests that HB's target food source be chosen as one of these food sources with the highest nectar refilling rate. After being noticed, this HB may fly in the direction of the ideal food supply.

These patterns of flight may be defined in *d*-D space, using the following axial flight definition:

$$F^{{(i)}} = \left\{ {\begin{array}{*{20}l} {1\, } & {\, if\, i = randi\left( {\left[ {1,d} \right]} \right)} \\ 0 & {else} \\ \end{array} } \right.\, \,\quad i = 1,2,...,d$$

The following is the definition of the diagonal flight:

$$F^{{(i)}} = \left\{ {\begin{array}{*{20}l} {1\, } & {if\, i = P(j),\, j \in \left[ {1,k} \right],\, P = randperm(k),\, k \in \left[ {2,\left[ {r_{1} .\left( {d - 2} \right)} \right] + 1} \right]} \\ 0 & {else} \\ \end{array} } \right.\, \,\quad i = 1,2,...,d$$

**Figure 2:**
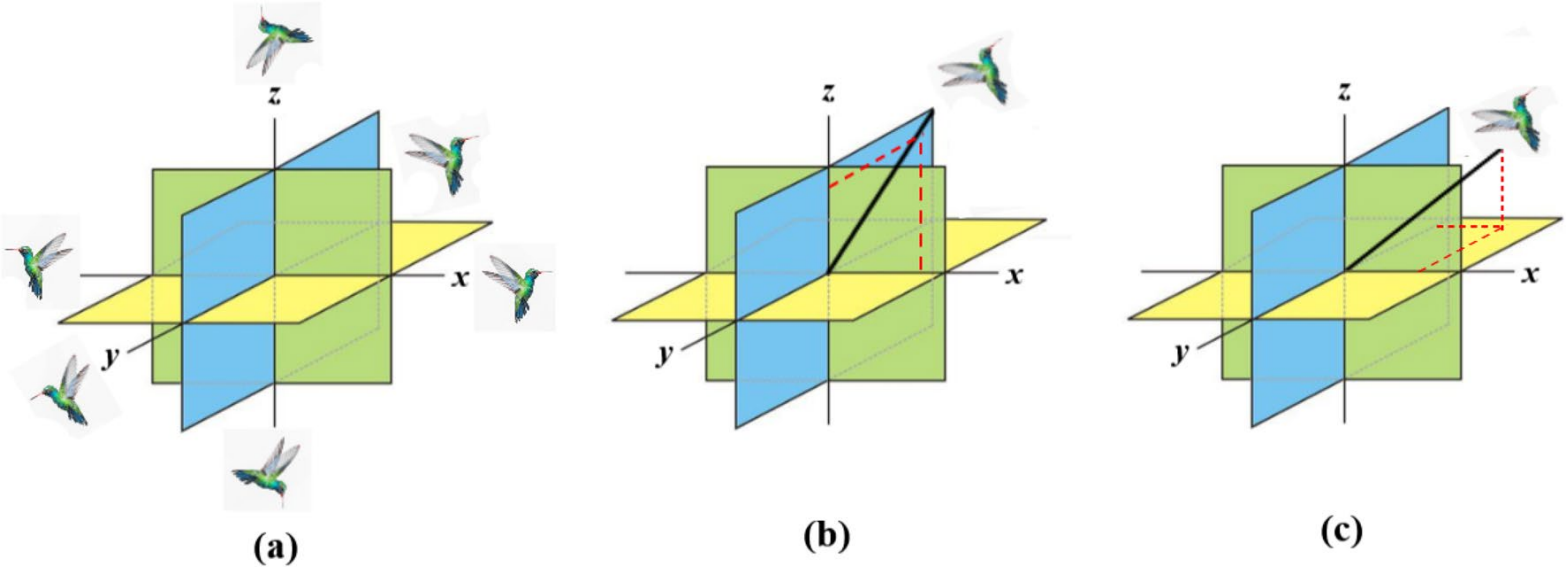
Flight behaviours of HBs: (**a**) axial, (**b**) diagonal, (**c**) omnidirectional flight (Zhao et al.^72^).

The following is the definition of omnidirectional flight:

$$F^{(i)} = 1\,\,i = 1,2,...,d$$

where randi([1, d]) generates an integer at random from 1 to *d*, *randperm*(*k*) generates a random permutation from 1 to *k*, and $r_{1}$ is a random number in (0, 1], and *randperm*(*k*) generates a random permutation from 1 to *k*. The diagonal flight in *d*-D space is enclosed within a hyper rectangle specified by any 2 to *d*-1 coordinate axes. Flight behaviours of HBs is presented in Fig. 2.

AHB's flying abilities enable it to visit its intended food source, leading in the acquisition of a potential food resource. As a result, a food source is updated. The (5) is used to simulate directed foraging activity and a probable food source:

17 $$w_{i} (t + 1) = u_{i,tar} (t) + c.F.\left( {u_{i} (t) - u_{i,tar} (t)} \right)$$

18 $$c \sim N\left( {0,1} \right)$$

If $u_{i} (t)$ represents the i-th nectar source at time *t*, $u_{i,tar} (t)$ represents the food source target that the i-th HB intends to visit, and an is a directed factor with a normal distribution. *N*(0,1) with a mean of 0 and a standard deviation of 1. Equation (17) discusses directed foraging by HBs employing different flight patterns by allowing each current source of food to update its location in relation to the target source of food.

19 $$u_{i} (t + 1) = \left\{ {\begin{array}{*{20}l} {u_{i} (t)\, \, } & {when\, f\left( {x_{i} \left( t \right)} \right) \le f\left( {w_{i} \left( {t + 1} \right)} \right)} \\ {w_{i} (t + 1)\, } & {when\, f\left( {x_{i} \left( t \right)} \right) > f\left( {w_{i} \left( {t + 1} \right)} \right)} \\ \end{array} } \right.$$

$f\left( {x_{i} \left( t \right)} \right)$ represents the function's fitness value. If the candidate food source's rate of nectar refilling is higher than the current one, the HB leaves the current food source and feeds at the food source that was found by (17), as stated in (18).

#### Territorial foraging

After consuming flower nectar from its target food source, an HB is less likely to visit its current food sources and is more likely to look for new ones. As a result, an HB might easily migrate to a nearby location inside its own zone where it might find a new food supply that is probably better than the existing one. The following mathematical formula can be used to predict how HB uses local foraging as part of their territorial strategy:

$$w_{i} (t + 1) = u_{i} (t) + e.F.u_{i} (t)$$

$$e \sim N\left( {0,1} \right)$$

where $e$ be the geographical component along with a mean of 0 and standard deviation of 1. It is to be noted that it follows normal distribution *N*(0,1). Based on the idea of a particular flying aptitude, every HB may discover a fresh supply of food in a search area that is in between. When the territorial foraging strategy is finished, the visit table will be updated.

#### Migration foraging

An HB will look for food in a more remote area when the food supply at their preferred feeding spot runs out. The AHA method makes use of a migration coefficient. If more iterations are needed than the migration coefficient, the HB at the food source with the lowest rate of nectar refilling will relocate to a new food source that is randomly generated throughout the search region. The new source will then be used in lieu of the previous one by this HB, and the visit table will update to reflect this. An HB's foraging migration from the source with the lowest nectar replenishment rate to a new one created at random can be explained by the following:

$$u_{wor} (t + 1) = lower + r\left( {upper - lower} \right)$$

where $u_{wor}$ is the population's weakest food source's rate of nectar replenishment.

The preference of HBs to migrate towards alternative food sources when there is no food source whose location is altered by the guided foraging strategy leads to more exploration and a decreased likelihood of convergence on a local optimal solution. When one food source is replaced by another, the new one is more likely to be exploited than the old since the same target source draws HBs from other food sources. According to (17), early in iterations, exploration is encouraged due to the great distance between food sources, but as iterations go on, the distance between food sources gets smaller adaptively, prioritising exploitation. An HB conducts the exploitation process in its immediate area by employing the territorial foraging approach. Additionally, HB movement foraging suggests that the HB has looked about the search area. Two often used parameters in AHA are the maximum number of repeats and population size, yet only one control parameter is necessary to determine whether migration happens. If there were no substitutes for any of the food sources, an HB would go to each one throughout each repetition. Let us assume that choosing between guided and territorial foraging has a 50% chance of success, and that during guided foraging, visiting every other source has a 50% chance of success. An HB can consider the same food supply to be the goal source after 2*n* repeats. In this case, migration must be used to broaden the search area and lessen stagnation. Therefore, it is suggested that the migration coefficient in terms of population size is *X* = *2n*.

In addition, to assess the effectiveness of AHA in solving (16), the following five well-known and well-reputed (in terms of efficiency) metaheuristics are used:

- Firefly Algorithm (FA) (Yang and He^76^)
- Grey Wolf Optimizer Algorithm (GWOA) (Mirjalili et al.^73^)
- Whale Optimization Algorithm (WOA) (Mirjalili and Lewis^77^)
- Artificial Electric Field Algorithm (AEFA) (Yadav^75^)
- Equilibrium Optimizer Algorithm (EOA) (Faramarzi et al.^74^)

## Numerical analysis

The average profit function $AP_{1} \left( {s_{p} ,\theta_{g} ,T} \right)$ is highly nonlinear with respect to the green level of the items, selling price and the cycle length. So, it is very problematic to explain analytically. Therefore, to validate and check the reality of our proposed model, we have considered one numerical example and solved it using Artificial Hummingbird Algorithm (AHA) (Zhao et al.^72^). Also, the optimal results obtained from AHA are compare to the optimal results obtained from Grey Wolf Optimizer Algorithm (GWOA) (Mirjalili et al.^73^), Equilibrium Optimizer Algorithm (EOA) (Faramarzi et al.^74^), Artificial Electric Field Algorithm (AEFA) (Yadav et al.^75^), Firefly Algorithm (FA) (Yang & He^76^) and Whale Optimizer Algorithm (WOA) (Mirjalili and Lewis^77^).

### Numerical example

In order to demonstrate the usefulness of the suggested production inventory model, we have considered a numerical example. The values of the parameters are taken hypothetically and it seems to be realistic.

#### Example 1.

The following data of the system parameters of our proposed model are considered.

$$\begin{gathered} d_{0} = 350\,unit;\,d_{1} = 3.5;\,d_{2} = 6.2;\,d_{3} = 5.9;\,d_{4} = 4.7;\,h_{c} = \$ 1.5;\,\lambda_{1} = \$ 15;\,\lambda_{2} = 0.35;\,\lambda_{3} = 0.65;\, \hfill \\ A = \$ 150;\,w_{c} = \$ 5;b_{1} = 1.5;a = 1.15;\,b_{2} = 2;\,c_{m} = \$ 1;\,\,b = 1.5;\,\,d = 1.9;\,c = 1.1;t_{w} = 12month; \hfill \\ \end{gathered}$$

The best-found and worst-found solutions of Example 1 obtained from AHA and others taken algorithms are presented In Tables 2, 3 respectively. Also, in Table 4, the statistical results are carried out, and the non -parametric test, i.e., analysis of variance, is shown in Table 5.

**Table 2:** Best-found solution obtained from AHA and other meta-heuristic algorithms of Example 1.

Algorithms	$$AP_{1}$$ ($/month)	$$s_{p}$$ ($/unit)	$$\theta_{g}$$	$$T$$ (month)	$${\text{Run}}\,{\text{time}}$$ (second)
AHA	${1171}.{991328}$	${65}.0{42366}$	$0.{883746}$	${16}.{8}0{4238}$	$0.{1}0{5998}$
AEFA	${1171}.{991328}$	${65}.0{42366}$	$0.{883746}$	${16}.{8}0{4239}$	$0.{447869}$
EOA	${1171}.{991328}$	${65}.0{42366}$	$0.{883746}$	${16}.{8}0{4239}$	$0.{2}0{5727}$
FA	${1171}.{991328}$	${65}.0{42288}$	$0.{883721}$	${16}.{8}0{4116}$	$0.{592856}$
GWOA	${1171}.{9913}0{3}$	${65}.0{42236}$	$0.{882534}$	${16}.{8}0{7318}$	$0.{179449}$
WOA	${1171}.{991311}$	${65}.0{42620}$	$0.{884754}$	${16}.{8}0{1797}$	$0.{123259}$

**Table 3:** Worst-found solution obtained from AHA and other meta-heuristic algorithms of Example 1.

Algorithms	$$AP_{1}$$($/month)	$$s_{p}$$($/unit)	$$\theta_{g}$$	$$T$$(month)	$${\text{Run}}\,{\text{time}}$$(second)
AHA	${1171}.{991328}$	${65}.0{42366}$	$0.{883746}$	${16}.{8}0{4239}$	$0.{124832}$
AEFA	${1171}.{961423}$	${64}.{952171}$	$0.{876111}$	${16}.{584598}$	$0.{377195}$
EOA	${1171}.{991328}$	${65}.0{42366}$	$0.{883746}$	${16}.{8}0{4239}$	$0.{284688}$
FA	${1171}.{991312}$	${65}.0{43600}$	$0.{884824}$	${16}.{8}0{4432}$	$0.{5}0{5}0{66}$
GWOA	${1171}.{989431}$	${65}.0{47340}$	$0.{875222}$	${16}.{843976}$	$0.{172271}$
WOA	${1171}.{953185}$	${64}.{993474}$	$0.{83}0{469}$	${16}.{8286}0{4}$	$0.{158515}$

**Table 4:** Results of statistical experiment of Example 1.

Algorithm	Best-found of $AP_{1}$	Worst-found of $AP_{1}$	Mean of $AP_{1}$	Mode of $AP_{1}$	Median of $AP_{1}$	Standard deviation
AHA	${1171}.{991328}$	${1171}.{991328}$	${1171}.{991328}$	${1171}.{991328}$	${1171}.{991328}$	${\mathbf{1}}{\mathbf{.60777 \times 10}}^{{{\mathbf{ - 12}}}}$
AEFA	${1171}.{991328}$	${1171}.{961423}$	${1171}.{987565}$	${1171}.{991328}$	${1171}.{99}0{798}$	$0.00{6748322}$
EOA	${1171}.{991328}$	${1171}.{991328}$	${1171}.{991328}$	${1171}.{991328}$	${1171}.{991328}$	${\mathbf{1}}{\mathbf{.60777 \times 10}}^{{{\mathbf{ - 12}}}}$
FA	${1171}.{991328}$	${1171}.{991312}$	${1171}.{991327}$	${1171}.{991328}$	${1171}.{991328}$	${3}.{17317} \times 10^{ - 06}$
GWOA	${1171}.{9913}0{3}$	${1171}.{989431}$	${1171}.{99}0{614}$	$-$	${1171}.{99}0{786}$	$0.000{6}00{573}$
WOA	${1171}.{991311}$	${1171}.{953185}$	${1171}.{98}0{358}$	$-$	${1171}.{9826}0{6}$	$0.0{1}0{8153}0{1}$

Bold face values are showing the performances of the algorithms in all respect.

**Table 5:** Analysis of variance (ANOVA) of Example 1.

AHA vs	Count	Average	Variance	Source of Variation						*F*	*F-crit*	*P-value*
				Between Groups			Within Group					
				*SS*	*df*	*MS*	*SS*	*df*	*MS*			
AEFA	$50$	${1171}.{987565}$	${4}.{55398} \times {10}^{ - 05}$	$0.{35} \times {10}^{ - 03}$	$1$	$0.{35} \times {10}^{ - 03}$	$0.{22} \times {10}^{ - 02}$	$98$	${2}.{27} \times {10}^{ - 05}$	$15.54$	${3}.{93}$	${\mathbf{0}}{\mathbf{.15 \times 10}}^{{{\mathbf{ - 03}}}}$
EOA	$50$	${1171}.{991328}$	${2}.{58494} \times {10}^{ - 24}$	${4}.{91} \times {10}^{ - 22}$	$1$	${4}.{91} \times {10}^{ - 22}$	${2}.{53} \times {10}^{ - 22}$	$98$	${2}.{58} \times {10}^{ - 24}$	${19}0$	${3}.{93}$	${\mathbf{1}}{\mathbf{.13 \times 10}}^{{{\mathbf{ - 24}}}}$
FA	$50$	${1171}.{991327}$	${1}.00{69} \times {10}^{ - 11}$	${3}.{48} \times {10}^{ - 11}$	$1$	${3}.{48} \times {10}^{ - 11}$	${4}.{93} \times {10}^{ - 10}$	$98$	${5}.0{3} \times {10}^{ - 12}$	${6}.{91}$	${3}.{93}$	${\mathbf{0}}{\mathbf{.99 \times 10}}^{{{\mathbf{ - 02}}}}$
GWOA	$50$	${1171}.{99}0{614}$	${3}.{6}0{68} \times {10}^{ - 07}$	${1}.{27} \times {10}^{ - 05}$	$1$	${1}.{27} \times {10}^{ - 05}$	${1}.{76} \times {10}^{ - 05}$	$98$	${1}.{8}0 \times 10^{ - 07}$	${7}0.{72}$	${3}.{93}$	${\mathbf{3}}{\mathbf{.35 \times 10}}^{{{\mathbf{ - 13}}}}$
WOA	$50$	${1171}.{98}0{358}$	$0.000{116971}$	$0.{3}0 \times 10^{ - 02}$	$1$	$0.{3}0 \times 10^{02}$	$0.{57} \times {10}^{ - 02}$	$98$	${5}.{84} \times {10}^{ - 05}$	${51}.{44}$	${3}.{93}$	${\mathbf{1}}{\mathbf{.41 \times 10}}^{{{\mathbf{ - 10}}}}$

Bold face values are showing the performances of the algorithms in all respect.

## Observations

- From Table 2, it is evident that the computational best-found solution of Example 1 obtained from AHA, AEFA, EOA and FA is equal to six decimal places analysed to the other results acquire from WOA and GWOA of the optimization Problem 1.
- Up to six decimal places, the computational best-found (cf. Table 2) and worst-found (cf. Table 3) solutions of Example-1 produced via AHA and EOA are identical. Additionally, Table 3 demonstrates that WOA provides the worst- value of Problem 1.
- Again, from Tables 2, 3, it seen that AHA takes minimum computational time to find the optimal solution of Example-1 than the other algorithms.
- It can be seen from Table 4 above that the standard deviations for AHA and EOA are the same and lowest. In light of the statistical analysis, it can be said that for Example 1, AHA and EOA provide more optimal results than the other algorithms.
- The F-static values for AHA, AEFA, EOA, FA, GWOA, and WOA are higher than the F-critical values, as shown by Table 5. So, null hypothesis is rejected in this case. Also, the $P$-values (boldfaced) of AHA versus AEFA, EOA, FA, WOA and GWOA are less than 0.05, for Example 1. Hence, due to ANOVA, AHA is significant statistically compared to AEFA, EOA, FA, GWOA, and WOA at a 5% significance level.

### Concavity graph of the average profit function

**Figure 3:**
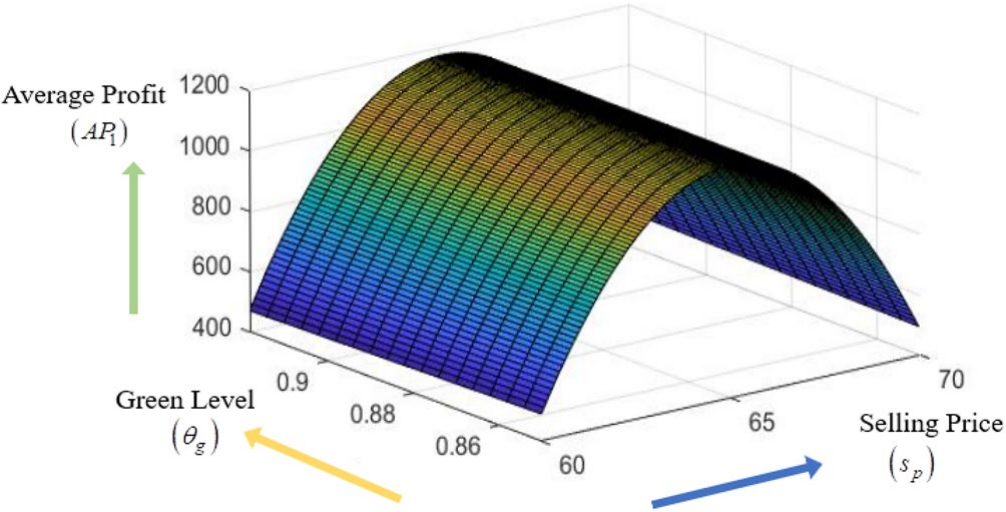
Concavity graph of $AP_{1}$ w.r.t. $\left( {s_{p} ,\theta_{g} } \right)$ of Example 1.

**Figure 4:**
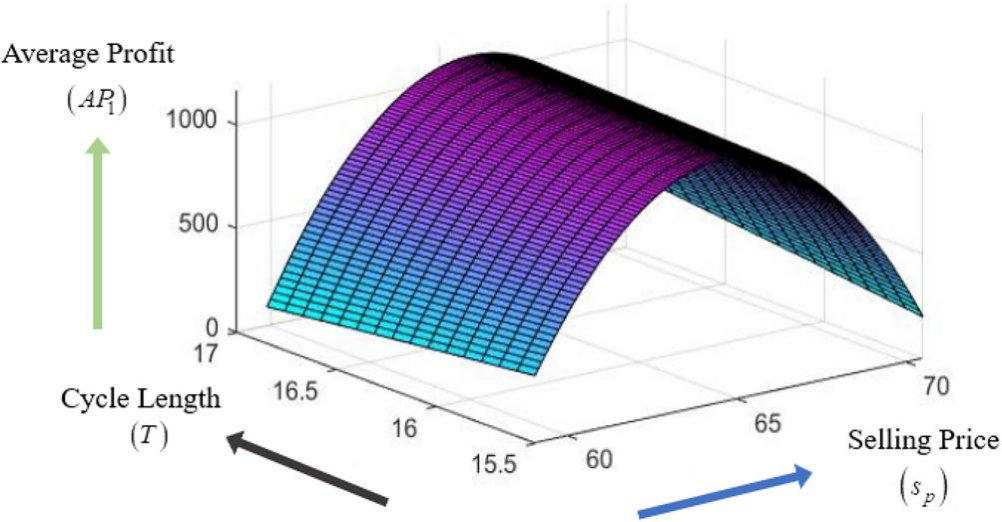
Concavity graph of $AP_{1}$ w.r.t. $\left( {s_{p} ,T} \right)$ of Example 1.

**Figure 5:**
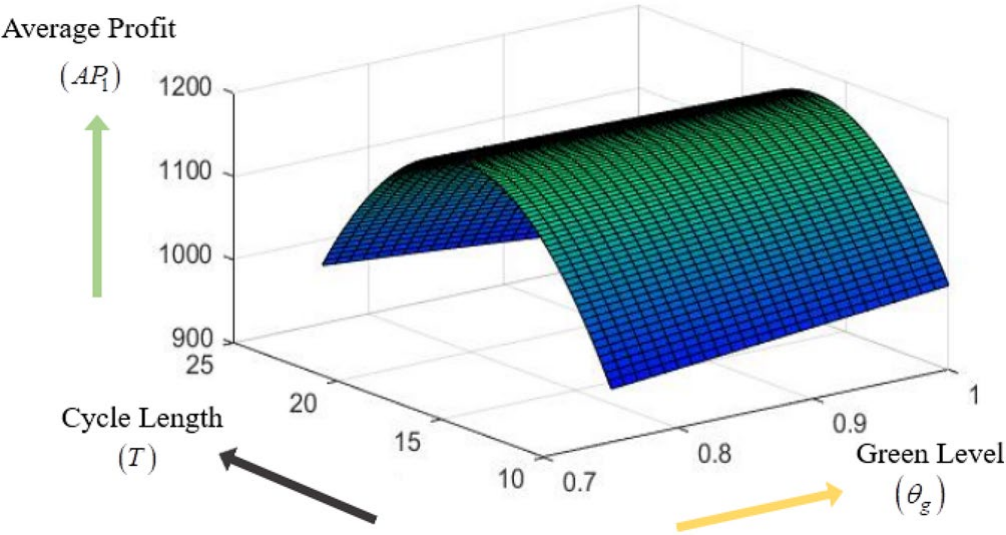
Concavity graph of $AP_{1}$ w.r.t. $\left( {T,\theta_{g} } \right)$ of Example 1.

The concavity of average profit function $AP_{1} \left( {s_{p} ,\theta_{g} ,T} \right)$ with respect to different decision variables $s_{p} ,\theta_{g} {\text{ and }}T$ are presented in the following Figs. 3, 4, 5.

### Convergence graph of metaheuristics algorithm

**Figure 6:**
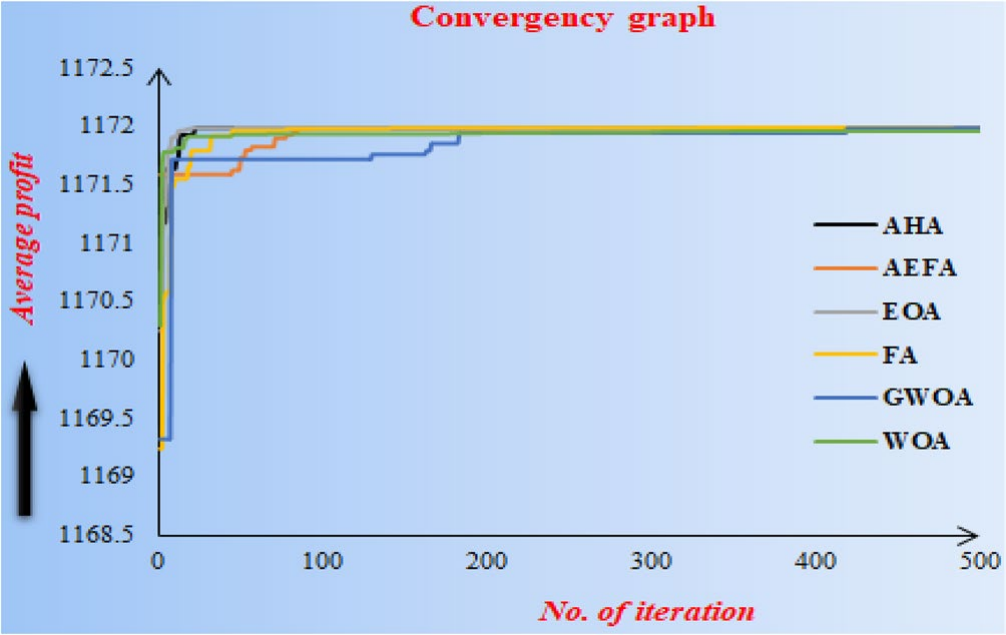
Convergence graph of different metaheuristics algorithms.

Convergence graph of different metaheuristics algorithms (viz. AHA, EOA, AEFA, GWOA, FA, WOA) to the average profit function $AP_{1} \left( {s_{p} ,\theta_{g} ,T} \right)$ of Example 1 are shown in the following Fig. 6**.**

From Fig. 6, it is concluded that GWOA converges slowly to solve the Example 1 whereas EOA, AHA and FA converge almost equally for solving Example 1.

## Sensitivity analysis

**Figure 7:**
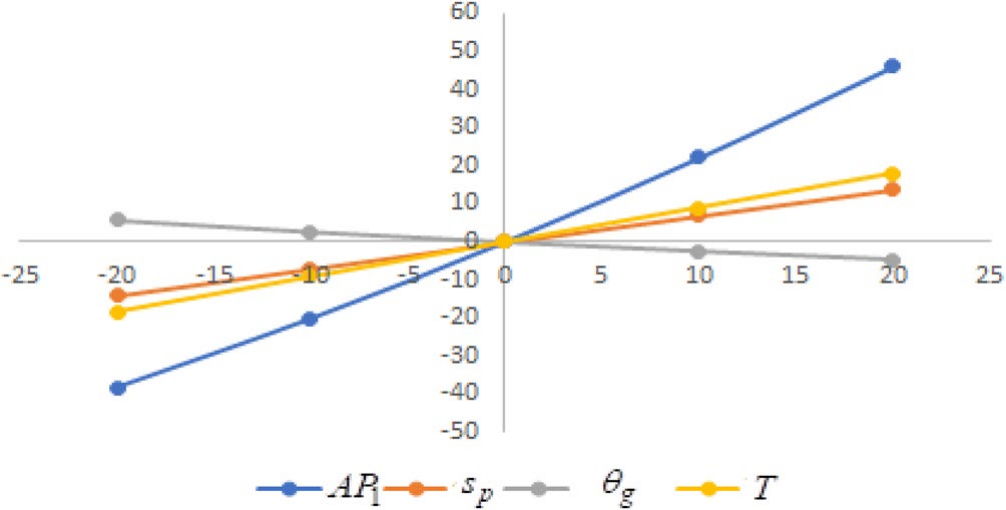
Effect of changes w.r.t. $d_{0}$.

**Figure 8:**
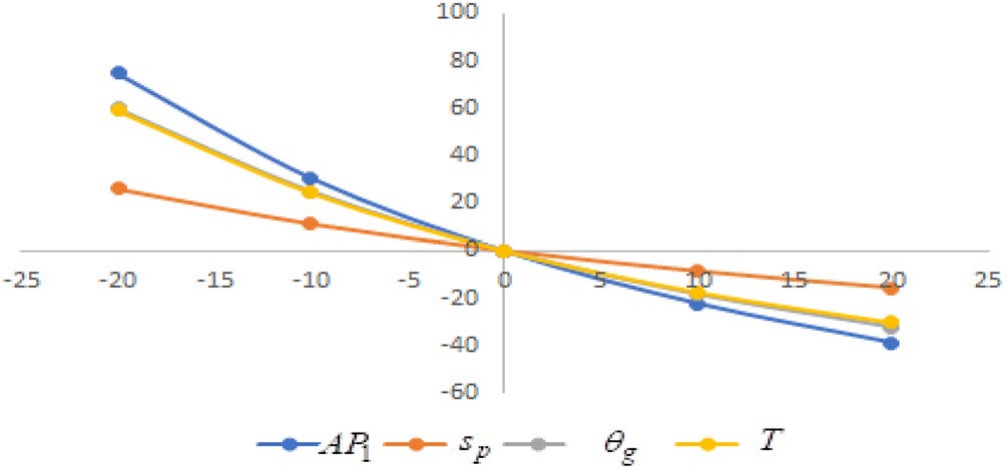
Effect of changes w.r.t. $d_{1}$.

**Figure 9:**
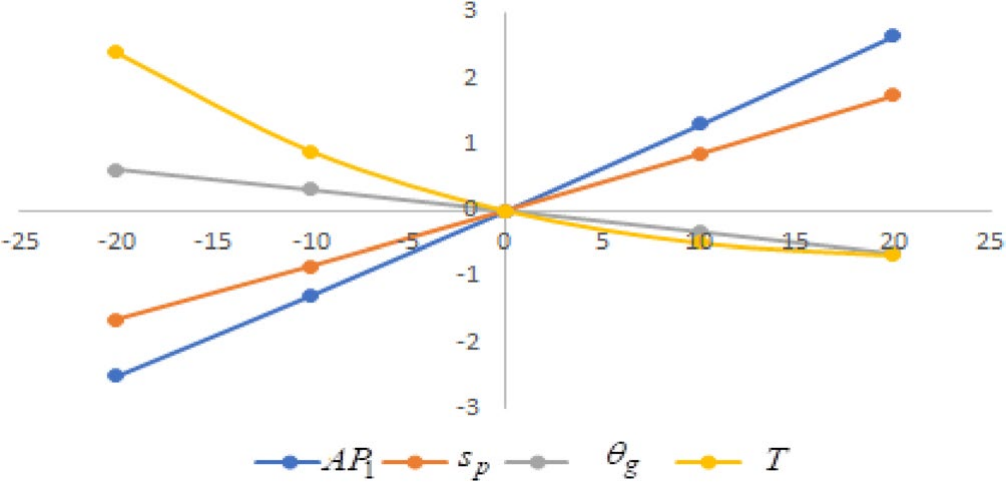
Effect of changes w.r.t. $d_{2}$.

**Figure 10:**
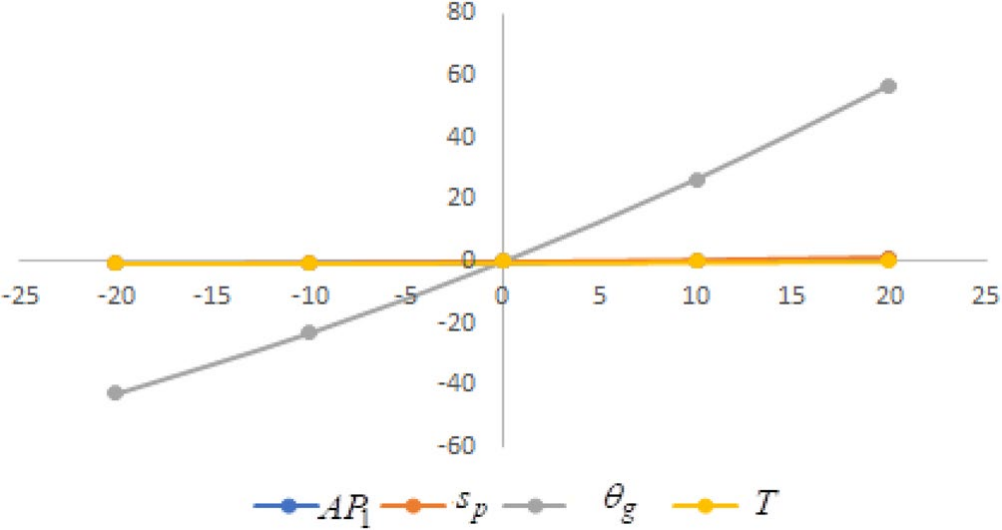
Effect of changes w.r.t. $d_{3}$.

**Figure 11:**
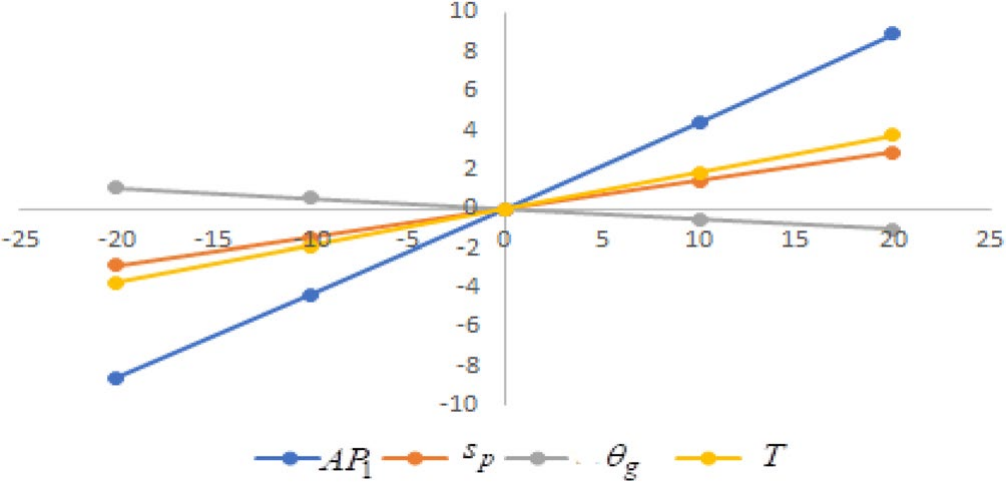
Effect of changes w.r.t. $d_{4}$.

**Figure 12:**
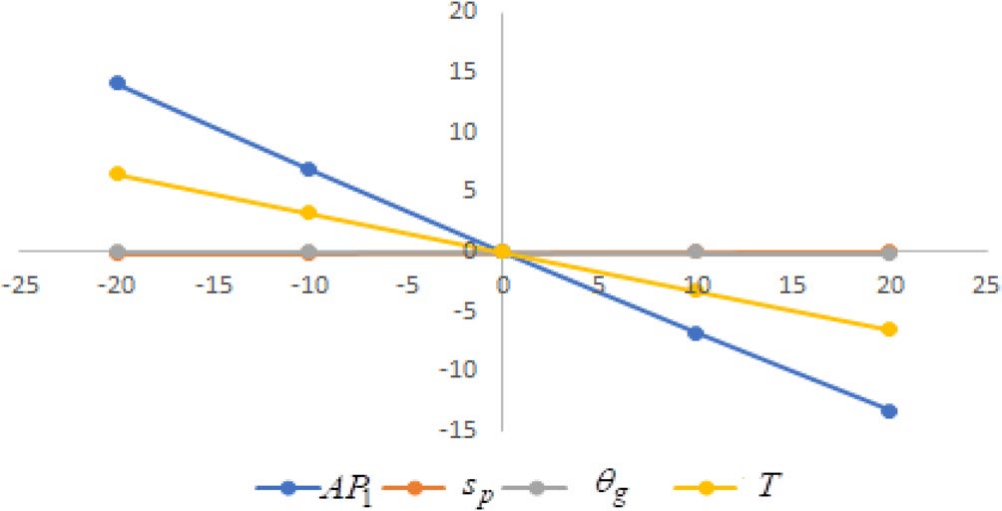
Effect of changes w.r.t. $\lambda_{1}$.

**Figure 13:**
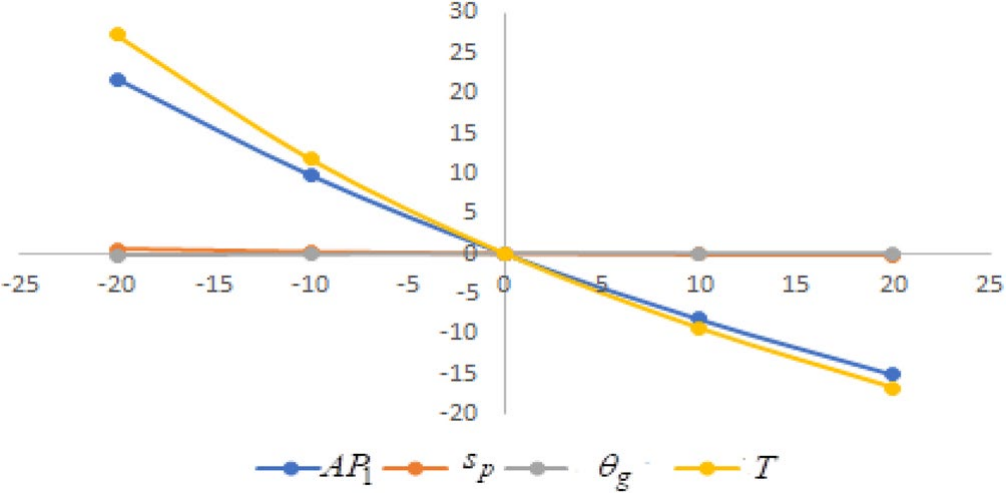
Effect of changes w.r.t. $\lambda_{2}$.

**Figure 14:**
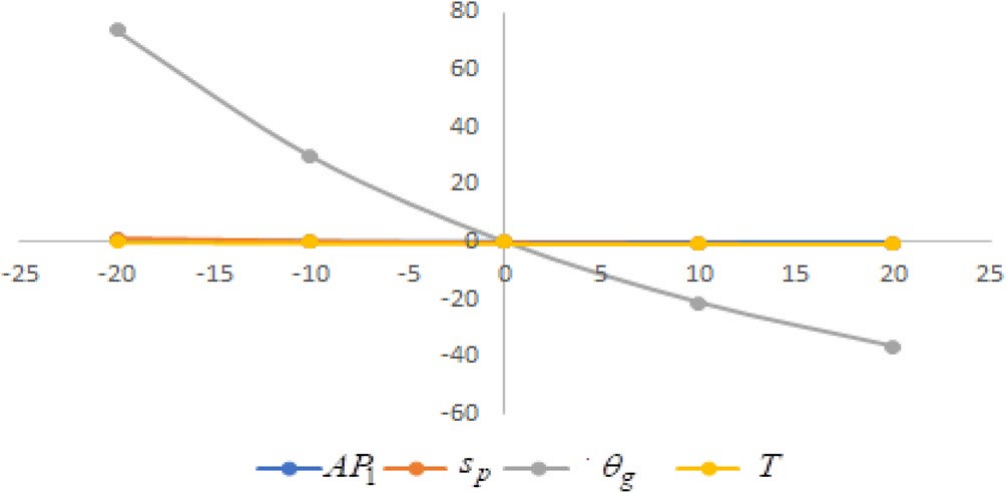
Effect of changes w.r.t. $\lambda_{3}$.

**Figure 15:**
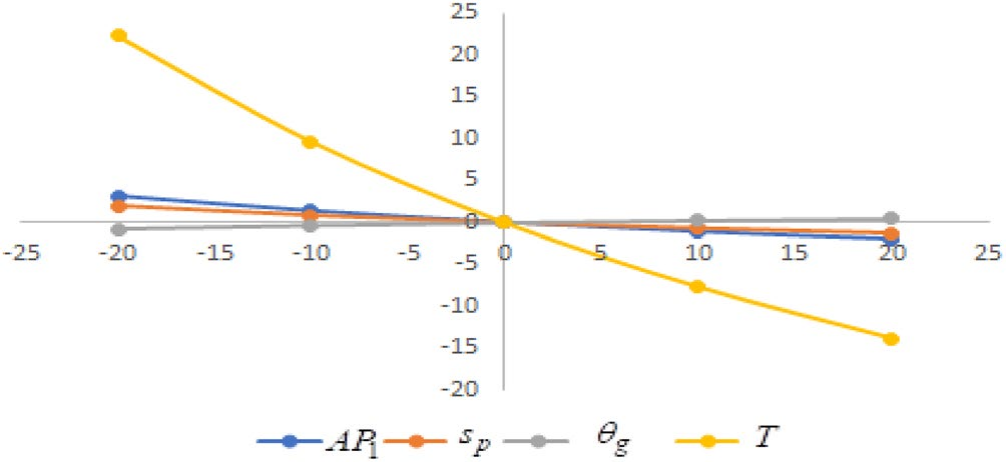
Effect of changes w.r.t. $h_{c}$.

**Figure 16:**
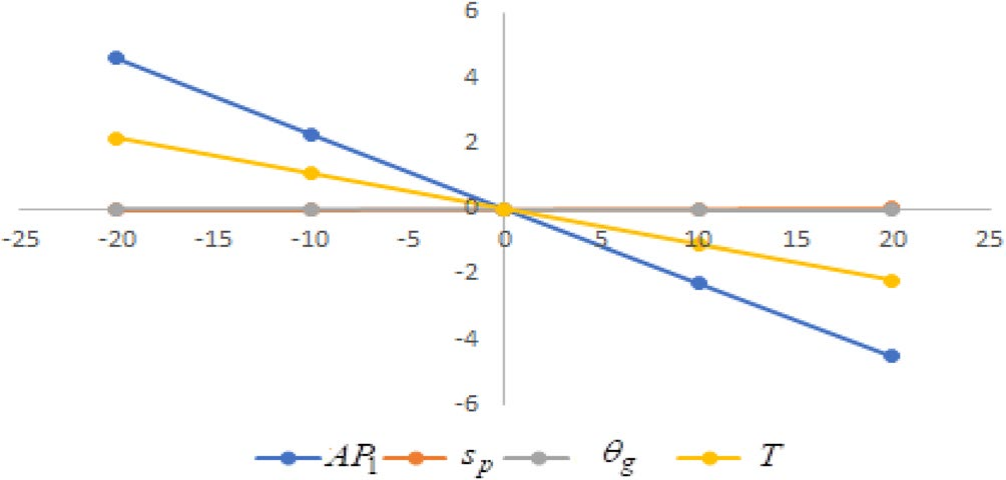
Effect of changes w.r.t. $w_{c}$.

**Figure 17:**
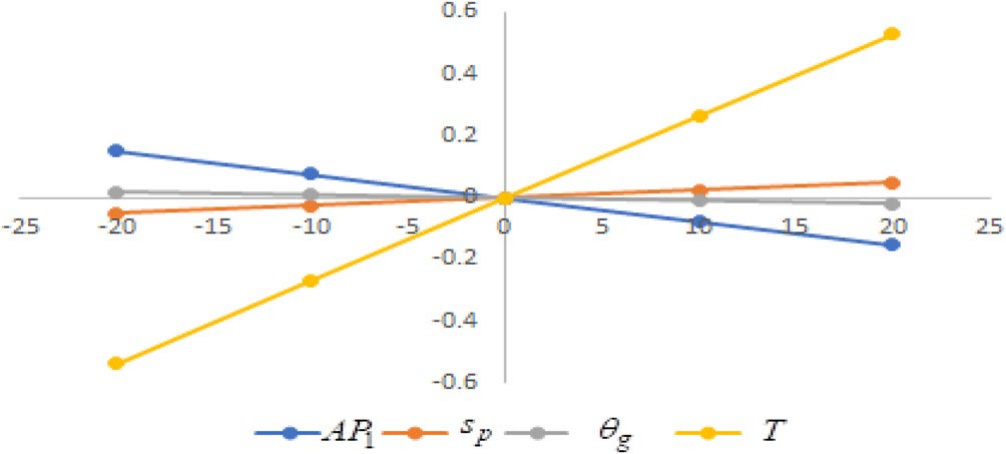
Effect of changes w.r.t. $A$.

**Figure 18:**
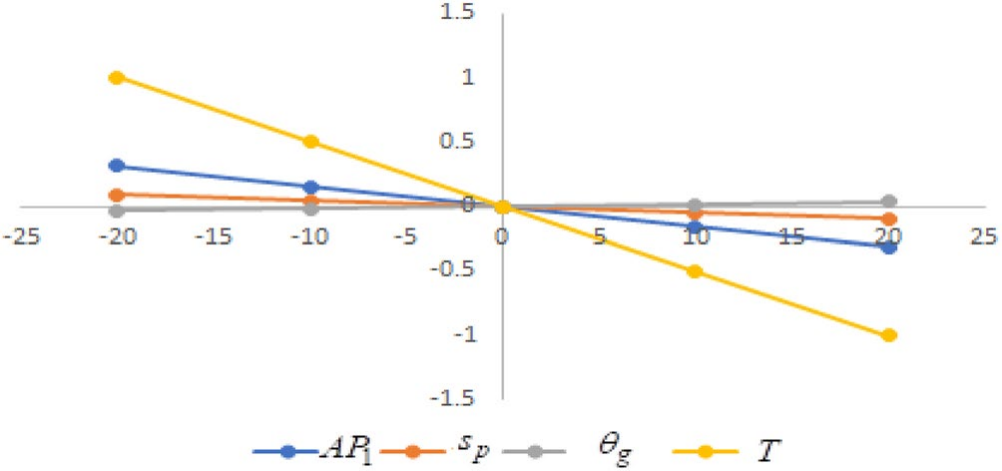
Effect of changes w.r.t. $c_{m}$.

In this section sensitivity analyses are carried out to show the impact of various proposed inventory system parameters on green level of the product $\left( {\theta_{g} } \right)$, selling price $\left( {s_{p} } \right)$, cycle length $\left( T \right)$ and average profit $\left( {AP_{1} } \right)$ for Example 1. In this experiment, each inventory parameter is changed by -20% to 20% at a time while the other parameters are left unchanged. Sensitivity analyses are presented in Figs. 7, 8, 9, 10, 11, 12, 13, 14, 15, 16, 17 and 18.

One can observe the following implications from the above sensitivity figures.

(i) $AP_{1} \left( {s_{p} ,\theta_{g} ,T} \right)$ is highly sensitive with the changes of initial fixed demand $\left( {d_{0} } \right)$ and parameter of the demand $\left( {d_{1} } \right)$.It is equally sensitive with respect to the production cost parameter $\left( {\lambda_{2} } \right)$.It is sensitive moderately against the changes of production cost parameter $\left( {\lambda_{1} } \right)$. Further, average profit is less sensitive to the demand parameters $\left( {d_{4} } \right)$ and per unit warranty cost $\left( {w_{c} } \right)$.Also, $AP_{1}$ is insensitive with respect to the demand parameters $\left( {d_{2} } \right),\left( {d_{3} } \right)$,production cost parameter $\left( {\lambda_{3} } \right)$, carbon emission investment parameters $\left( {c_{m} } \right)$, per unit holding cost $\left( {h_{c} } \right)$, and set-up cost $\left( A \right)$. Also, the parameter $\left( {d_{1} } \right)$ has a reverse effect on the average profit.
(ii) The selling price $\left( {s_{p} } \right)$ is largely effective for the demand parameter $\left( {d_{1} } \right)$ whereas, It is sensitive with moderately respect to $\left( {d_{0} } \right)$. Further, the demand parameter $\left( {d_{2} } \right),\left( {d_{3} } \right)\,{\text{and}}\left( {d_{4} } \right)$, production cost parameter $\left( {\lambda_{1} } \right),\left( {\lambda_{2} } \right)\,{\text{and}}\,\left( {\lambda_{3} } \right)$, per unit warranty cost $\left( {w_{c} } \right)$, carbon emission investment parameter $\left( {c_{m} } \right)$, per unit holding cost $\left( {h_{c} } \right)$, and set-up cost $\left( A \right)$ have no effect on $\left( {s_{p} } \right)$. Also, the parameter $\left( {d_{1} } \right)$ have a reverse effect on $\left( {s_{p} } \right)$.
(iii) From the Figs. 7, 8, 9, 10, 11, 12, 13, 14, 15, 16, 17 and 18, it is seen that the green level of the product $\left( {\theta_{g} } \right)$ is highly sensitive with respect to $\left( {d_{1} } \right)\,,\left( {d_{3} } \right)$ and for the production cost parameter $\left( {\lambda_{3} } \right)$. It is less sensitive against the changes of $\left( {d_{0} } \right).$ Further, $\left( {\theta_{g} } \right)$ is insensitive to the demand parameters $\left( {d_{2} } \right),\left( {d_{4} } \right)$, production cost parameter $\left( {\lambda_{1} } \right),\left( {\lambda_{2} } \right)$, per unit warranty cost $\left( {w_{c} } \right)$, carbon emission investment parameter $\left( {c_{m} } \right)$, per unit holding cost $\left( {h_{c} } \right)$, and set-up cost $\left( A \right)$. Also, the parameters $\left( {d_{1} } \right)\,{\text{and}}\,\left( {\lambda_{3} } \right)$ have reverse effect on $\left( {\theta_{g} } \right)$.
(iv) The cycle length $\left( T \right)$ is highly sensitive with the changes of $\left( {d_{1} } \right)$,production cost parameter $\left( {\lambda_{2} } \right)$ and per unit holding cost $\left( {h_{c} } \right)$.It is sensitive moderately against the changes of $\left( {d_{0} } \right).$ The cycle length is less sensitive with respect to $\left( {\lambda_{1} } \right)$. Further, $\left( T \right)$ is insensitive with respect to $\left( {d_{2} } \right),\left( {d_{3} } \right),\left( {d_{4} } \right)$, production cost parameter $\left( {\lambda_{3} } \right)$, per unit warranty cost $\left( {w_{c} } \right)$, carbon emission investment parameter $\left( {c_{m} } \right)$, per unit holding cost $\left( {h_{c} } \right)$, and set-up cost $\left( A \right)$. Also, the parameter $\left( {d_{1} } \right)$ has a reverse effect on the cycle length.

## Managerial insights

Warranty and greenness of products play a key role in product management and customer service in order to satisfy customer demand. It entails being aware of the management implications of product warranties and the greenness of the product. Here some managerial insight given below:

- A comprehensive guarantee demonstrates your assurance regarding the level of quality of your merchandise. Because of the product's high quality, customers' confidence and loyalty may grow. Customers who think a product is of higher quality and worth are more likely to acquire it if its warranty is longer.
- Managers need to consider how to strike a balance between the advantages of warranties and the costs involved. This includes the cost of customer support, the cost of repairs or replacements, and the impact on profitability. By examining customer feedback pertaining to warranties, warranty lengths and coverage can be optimised.
- Customer feedback can offer insightful information about how well a product performs in the using condition. Managers can utilise warranty information to spot recurrent problems and gradually raise product quality.
- Because of the benefits, they have for the environment; green products frequently have greater perceived values. Customers might be prepared to spend more on goods that reflect their values of sustainability and social responsibility.
- The green attributes of a product can make it stand out from rivals and develop a distinctive marketing proposition. Customers who care about the environment may purchase the goods if it provides real environmental advantages.
- Managers need to calculate the expenses of making a product that is more ecologically friendly. Green materials, manufacturing techniques, and certifications can affect production costs. Pricing choices must consider these costs.

## Concluding remarks and future scope

This study examined how warranty policies, carbon taxes, and green manufacturing affect a manufacturing system. This study also examines how consumers' demand and the average profit margin of the manufacturer are affected by factors such as warranty duration, pricing, and product greenness. In this context, the AHA, AEFA, EOA, FA, GWOA, and WOA algorithms are used to a maximising issue corresponding to the manufacturer's average profit. A sensitivity analysis and numerical experiment are taken into consideration to support the suggested production inventory model. A statistical test is also used to examine the effectiveness and performance of the AHA, AEFA, EOA, FA, GWOA, and WOA algorithms using the calculated results of the aforementioned maximisation problem. The research yields the following conclusions:

- After the product's green level and warranty are extended, the model will be more cost-effective for the manufacturer businesses.
- Policies that collect carbon taxes and promote the production of green products can reduce pollution in the environment, benefiting both manufacturing companies and the environment in a sustainable manner.
- Compared to other metaheuristic algorithms (AEFA, EOA, FA, GWOA, and WOA), AHA produces superior best-found outcomes. Therefore, compared to other algorithms taken into consideration in this work, the performance of AHA is more significant.

It is possible to conclude by saying that every industrial industry can use our suggested model. Those manufacturing companies will create environmentally friendly products and provide the customer with a warranty.

Potential avenues for further research include investigating uncertain (fuzzy, interval, and stochastic) deterioration or faulty production. Stock, advertisement-sensitive demand, power-type demand, etc. are further ways it might be expanded. In addition, for further extension, this model can incorporate advanced and delayed payment principles.

The main limitation of this model is unable to find the close form solution of the objective function due to high nonlinear of the objective function. To overcome this difficulty, we have used numerical technique for solving such type of optimization/optimal control problem.

## Data Availability

The datasets used and/or analysed during the current study available from the corresponding author on reasonable request. We don’t used any published data for solving the optimization problem.

## List of symbols

$$M\left( t \right)$$: Manufacturer production rate at time $$t$$
$$d_{0}$$: Initial market demand
$$d_{1} ,d_{2} ,d_{3} ,d_{4}$$: Parameters of demand rate
$$D_{1} \left( {s_{p} ,t,\theta_{g} ,t_{w} } \right)$$: Customers’ demand rate of the items
$$x\left( t \right)$$: Inventory level of manufacturer at time $$t$$
$$\theta_{m} \left( t \right)$$: Carbon emission rate at time $$t$$(ton/time unit)
$$c_{m}$$: Government payment of a carbon emission tax per unit ($/time unit)
$$\lambda_{1}$$: First-time production expenses ($/unit)
$$\lambda_{2} ,\lambda_{3}$$: Parameters of production costs
$$c_{p} \left( {M\left( t \right),\theta_{g} } \right)$$: Manufacturer per unit production cost of the items($/unit)
$$h_{c}$$: Per unit holding cost of the inventory($/unit)
$$w_{c}$$: Warranty cost of demand product ($/unit)
$$A$$: Set-up cost ($/cycle)
$$\theta_{g}$$: Green level of the product (Decision variable)
$$t_{w}$$: Warranty time of the product (time unit)
$$s_{p}$$: Selling price of the items ($/unit) (Decision variable)
$$T$$: Cycle length (time unit) (Decision variable)
$$AP_{1} \left( {s_{p} ,\theta_{g} ,T} \right)$$: Average profit function ($/time unit)
